# The Role of Glucose, Insulin and Body Fat in Assessment of Bone Mineral Density and Trabecular Bone Score in Women with Functional Hypothalamic Amenorrhea

**DOI:** 10.3390/jcm13154388

**Published:** 2024-07-26

**Authors:** Elżbieta Sowińska-Przepiera, Mariola Krzyścin, Igor Syrenicz, Adrianna Orlińska, Adrianna Ćwiertnia, Adam Przepiera, Karolina Jezierska, Aneta Cymbaluk-Płoska, Žana Bumbulienė, Anheli Syrenicz

**Affiliations:** 1 Pediatric, Adolescent Gynecology Clinic, Department of Gynecology, Endocrinology and Gynecological Oncology, Pomeranian Medical University in Szczecin, Unii Lubelskiej 1, 71-252 Szczecin, Poland; elzbieta.sowinska.przepiera@pum.edu.pl; 2 Department of Endocrinology, Metabolic and Internal Diseases, Pomeranian Medical University in Szczecin, Unii Lubelskiej 1, 71-252 Szczecin, Poland; igor.syrenicz@gmail.com (I.S.); anhelli.syrenicz@pum.edu.pl (A.S.); 3 Department of Reconstructive Surgery and Gynecological Oncology, Pomeranian Medical University in Szczecin, Al. Powstańców Wielkopolskich 72, 70-111 Szczecin, Poland; adzahorowska@gmail.com (A.O.); aneta.cymbaluk@gmail.com (A.C.-P.); 4 Department of Urology and Urologic Oncology, Pomeranian Medical University in Szczecin, Al. Powstańców Wielkopolskich 72, 70-111 Szczecin, Poland; przepiera@gmail.com; 5 Department of Medical Physics, Pomeranian Medical University, ul. Ku Słońcu 13, 71-073 Szczecin, Poland; karolina.jezierska@pum.edu.pl; 6 Clinics of Obstetrics and Gynecology, Institute of Clinical Medicine, Faculty of Medicine, Vilnius University, LT-08661 Vilnius, Lithuania; zana.bumbuliene@mf.vu; 7 Centre of Obstetrics and Gynecology, Vilnius University Hospital Santaros Klinikos, LT-08661 Vilnius, Lithuania

**Keywords:** trabecular bone score, bone mineral density, adipose tissue, glucose, insulin, osteoporosis, functional hypothalamic amenorrhea

## Abstract

**Background:** For years, bone mineral density (BMD) has played a key role in assessing bone health, but the trabecular bone score (TBS) is emerging as an equivalent measure. However, BMD alone may not fully measure bone quality or predict osteoporosis risk. To evaluate the usefulness of TBS and BMD in estimating the risk of bone fracture in young women with FHA, this study examined the association between metabolic parameters and bone quality, which was measured using TBS and BMD. **Methods:** We analyzed the association of metabolic factors with tests assessing bone quality—TBS and BMD. Patients were checked for BMI, measured body fat, and determined serum glucose levels and insulin levels in a 75g glucose load test. Spearman correlation analysis was used. **Results:** Significant positive correlations were found between BMD and age (*p* < 0.001) and body fat (*p* < 0.001), as well as between TBS values and BMI (*p* < 0.001) and TBS and percent body fat (*p* < 0.001). Of the variables analyzed in the multivariate analysis, the only independent predictor of higher bone mineral density in the lumbar spine was found to be higher values of the trabecular bone index in the same segment (*p* < 0.001). **Conclusions:** The use of TBS provides a simple tool for estimating the risk of bone damage. Ultimately, early screening, diagnosis and treatment of patients with FHA may help prevent osteoporosis and fragility fractures in the long term.

## 1. Introduction

Bone mineral density (BMD) has long been used to assess bone quality, determined by dual-energy X-ray absorptiometry (DEXA). DEXA uses collimated X-ray beams that pass through the tissue of the patient’s body and then are recorded by the detector. DEXA’s basic method is to measure the attenuation of X-ray radiation as it passes through the body part being studied and compare the results to the beam’s intensity from standard phantoms with known densities. The basic principle of DEXA is to measure the attenuation of x-ray energy during passing through the examined body area and compare results with the intensity of the beam coming from standard phantoms of known density [1]. DEXA measurements are used to monitor such parameters as changes in BMD, the gold standard in the diagnosis of osteoporosis or one of the most widely used assessments of bone microarchitectural texture quality—trabecular bone score (TBS) [2]. DEXA does expose the operator and patient to ionizing radiation but the absorbed dose in both cases is very small [3]. Recent studies suggest that BMD determined by this method is not an independent predictor of osteoporotic fractures. Moreover, this parameter may be inaccurate in individuals with extremely low or high body mass [4,5]. Some studies suggest that individuals with type 2 diabetes may have an increased risk of fractures due to low bone turnover and poorer microarchitecture, despite preserved BMD. Persons with diabetes have reduced levels of serum osteocalcin and C-terminal cross-linked telopeptide (CTX). These individuals may have reduced spongy bone volume, cortical layer thickness, lower osteoid volume, and osteoid thickness. These findings suggest a role for osteocalcin and CTX in bone formation in people with diabetes. There is also evidence that osteocalcin could play an important role in the treatment of diabetes [6,7]. These findings have led to the development of a more accurate marker of trabecular bone microarchitecture, digitally extracted from densitometric images [8]. Previous studies using TBS, many of which focused on diverse populations, have shown that this methodology can predict fracture risk in adults with low BMD or poor bone quality [9,10,11,12]. TBS is used to assess the structural build of bones, with a low TBS indicating compromised bone microarchitecture and serving as a predictor of osteoporotic fractures. It is also partially independent of clinical risk factors such as type 2 diabetes, chronic excess corticosteroids, and other conditions where BMD readings are often misleading [13]. This indicator may prove to be suitable for detecting bone fragility associated, among other factors, with obesity and type 2 diabetes. Lifestyle has a strong correlation with obesity and bone quality. In order to take care of bone health, moderate to higher intensity sports are recommended. Particular emphasis should be placed on this aspect in Brazil, Germany and China, as people in these countries have a higher incidence of diabetes, which also affects bone quality [14].

Smoking is also not without significance. A systematic review by Al-Bashaireh et al. analysed 243 articles focusing on the effects of tobacco on the musculoskeletal system. The majority of the studies indicate a negative contribution from smoking, highlighting lower BMD among smokers. Thus, smokers may have an increased risk of fractures, joint disease, ligament, cartilage and muscle deterioration [15].

The COVID-19 pandemic began in 2020 and has left its mark on the health of many people. Infection with the virus has also been linked to bone health. In a study of 773 adult Slovakians, a significant impact of the COVID-19 pandemic on bone tissue was observed [16]. Further studies in adult populations have assessed the utility of combined TBS and BMD assessment to enhance fracture risk prediction [17,18,19]. Monitoring bone quality, although not required in every patient, is very important in a group of women with functional hypothalamic amenorrhea (FHA). FHA is a common cause of amenorrhoea in adolescence. Prolonged FHA can have metabolic, cardiovascular, mental, reproductive and bone health implications. One of the most significant complications is loss of bone mass. Some patients with this disorder may develop osteoporosis, especially stress fractures. This is due to low bone mass and predominant resorption [20]. Inclusion criteria for such patients for DEXA testing include >6 months of amenorrhoea and the presence of a major weight loss or stress fracture. If bone mineral density is low, vitamin D deficiency should be ruled out by determining 25-hydroxyvitamin D levels. A group of women in whom monitoring by DEXA scanning is recommended are those with chronic FHA. This population should undergo DEXA scanning every 1 to 2 years [21]. Furthermore, women with functional hypothalamic amenorrhoea emphasise stress sensitivity and increased metabolic and hormonal responses associated with exercise. In a study Sanders’ et al. observed an increased cortisol response to exercise in women with functional amenorrhoea and a greater decrease in glucose levels than in women without menstrual disorders. Bone quality is affected by both low body fat mass and high body fat mass [22]. Obesity and insulin resistance are factors that may increase the risk of osteoporosis [23]. Until recently, earlier research results suggested a positive correlation between body mass and bone mineralization, attributing this connection to the stimulating effect of increased mechanical load on osteogenesis [24]. This belief was supported by the notion that mechanical load stimulates bone formation by reducing apoptosis and increasing the proliferation and differentiation of osteoblasts and osteocytes through the Wnt/β-catenin signaling pathway [25,26,27]. Therefore, it was thought that obesity might prevent bone loss and osteoporosis [28,29]. However, subsequent studies revealed that bone mineralization is influenced by fat mass rather than total body weight or BMI (body mass index) [30,31]. Current research suggests that obesity may increase the risk of certain fractures due to the existence of multiple pathways between adipose tissue and bones. Leptin, adiponectin, adipocytic estrogens, as well as insulin and amylin, may be involved in these connections [32]. Visceral adipose tissue and insulin resistance can influence bone mineralization independently of mechanical or hormonal effects resulting from excess adipose tissue [33]. However, adipose tissue serves as a source of pro-inflammatory cytokines, such as interleukin 6 (IL-6) and tumor necrosis factor α (TNF-α), oxidized low-density lipoprotein cholesterol molecules, and excess free fatty acids—all of which promote bone resorption. Diabetes, along with obesity, is associated with bone marrow adipogenesis, depriving mesenchymal stem cells available for osteoblast formation [34,35].

This article aims to evaluate the role of the above markers to assess bone quality expressed in both TBS and BMD in a population of young women diagnosed with FHA. By determining the role of BMD and TBS in assessing bone quality, as well as the influence of other factors, the results of this study may help determine the risk of osteoporosis in women with FHA.

## 2. Materials and Methods

### 2.1. Participation in the Study

The study included 213 women aged 20 to 33 years examined in 2015–2017. The following were the inclusion criteria for the study: Caucasian race; first menstruation between the ages of 12 and 13; episodes of secondary amenorrhea lasting three to six months in the previous year; transient psychological issues related to school, family, or work; female patients not receiving long-term medication. The following endocrinopathies were excluded: diagnosed by history, gynaecological examination, laboratory tests and endocrinopathies affecting bone mineralisation. In addition, a history of the following disorders was excluded: low birth weight, prematurity, nutritional disorders, abnormal nutrition during childhood and/or adolescence, growth and weight gain disorders, intensive sports participation metabolic diseases, use of stimulants and drugs affecting bone metabolism and processes, and a positive family history of bone quality disorders. After analyzing all of the above factors, it was concluded that this group of patients had a psychogenic type of menstrual disorder functional hypothalamic amenorrhea (FHA), which is diagnosed by exclusion.

The study was approved by the Bioethics Committee of the Pomeranian Medical University, number KB-0012/115/15.

### 2.2. Research Methodology

#### 2.2.1. Anthropometric Measurements and Gynaecological Examination

All patients had their anthropometric measurements—height [cm], weight [kg], adipose tissue and body mass index [kg/m^2^] (BMI)—calculated following a history and physical examination. BMI is the weight in kilograms divided by the square of the height in meters. Adipose tissue content was measured using the Bioelectrical Impedance Analysis (BIA). 

#### 2.2.2. Laboratory Parameters

In this study, the following were also determined glucose and insulin concentrations—baseline and 60′ and 120′ after the 75 glucose administration. During the patient’s stay at the Clinic, each patient had blood collection, a sample of which was forwarded to the diagnostic laboratory. Glucose, insulin levels were then determined. During the 75 g glucose test, the patient’s blood was drawn at 0′, then the patient was given a solution with 75 g glucose, and again after 60′ and 120′ minutes, blood was drawn and glucose and insulin were determined.

#### 2.2.3. Bone Mineral Density Assessment

Bone mineral density testing of the L1–L4 segment of the lumbar spine and the entire skeleton was performed on all study participants. Collimated X-ray beams were used in DEXA, where they enter the patient’s body through tissue and are subsequently detected by a detector. The fundamental idea behind DEXA is to determine how much X-ray energy is attenuated as it passes through the body part being studied and compare the results to the beam intensity from standard phantoms with established densities.

Due to the young age of the female patients (20–33 years), a Z-score was checked in the study group. Z-scores, which compare a young woman’s BMD to an age, gender, and ethnicity-matched population, are recommended by the International Society for Clinical Densitometry (ISCD) despite the similarities between T- and Z-scores in young individuals. In young women, a BMD Z-score of ≤2.0 was deemed by the ISCD to be below the predicted range for their age. Additionally, they advised interpreting BMD measurements in premenopausal females using two age ranges: before and after the age of PBM [36].

BMD was determined by DEXA (GE Lunar Prodigy Advance, Madison, WI, USA; enCORE version 8.8 software). Results are presented as absolute values (g/cm^2^).

Assessment of the microarchitecture of bone beads TBS values of the same lumbar vertebra were determined from DEXA images using analysis software (TBS INsight, version 2.1.2.0, Medimaps, Mérignac, France).

### 2.3. Statistical Analysis

The normality of the distribution of continuous variables was verified using the Shapiro-Wilk test. Statistical characteristics of continuous variables were presented in the form of arithmetic means, standard deviations (SD), medians, lower and upper quartile values and extreme values (min. and max.). 

The strength and direction of the relationship between pairs of continuous variables were assessed based on Spearman’s rank correlation coefficient (R) values. Parameters that showed a statistically significant (*p* ≤ 0.05) or close to statistical significance (*p* ≤ 0.1) relationship with the dependent variables (lumbar spine bone mineral density or lumbar spine trabecular bone score) were subjected to multiple regression analysis to identify independent predictors of bone mineral density. 

During multivariate regression analysis, beta values were calculated along with their standard error, as well as the model’s coefficients of determination (R2) along with their *p*-values. Parameters with *p*-values ≤ 0.05 were considered independent predictors of bone mineral density. All calculations were performed using Statistica 10 software (StatSoft, Tulsa, OK, USA).

## 3. Results

### 3.1. Characteristics of the Group

The study included 213 women between the ages of 20 and 33. The study group included 108 (50.7%) normal-weight women, 12 (5.6%) underweight, 38 (17.8%) overweight and 55 (25.8%) obese. Detailed statistical characteristics of the patient’s age and their body mass index are described below.

Mean age was 27.08, SD = 4.33, and the median was 27.00. The Mean BMI kg/m^2^ was 25.60, 5.82 SD and the median was 23.80 (Table 1).

**Figure 1:**
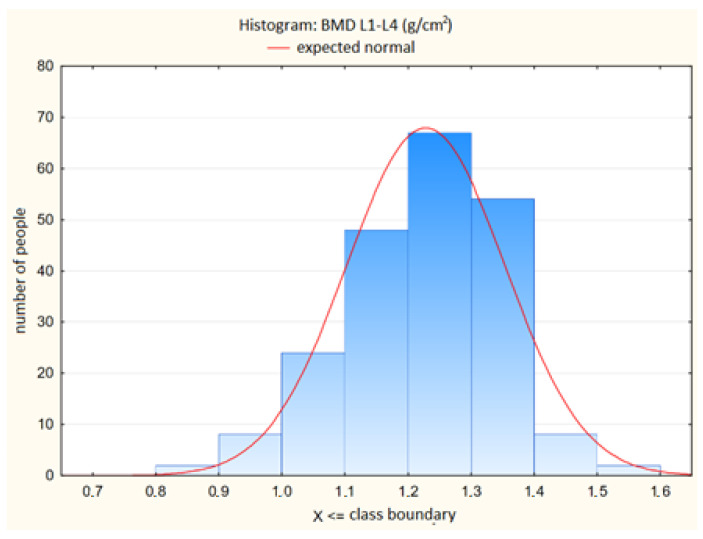
Distribution of Bone Mineral Density (BMD L1—L4, g/cm^2^) in the Lumbar Spine Among Study Participants.

**Figure 2:**
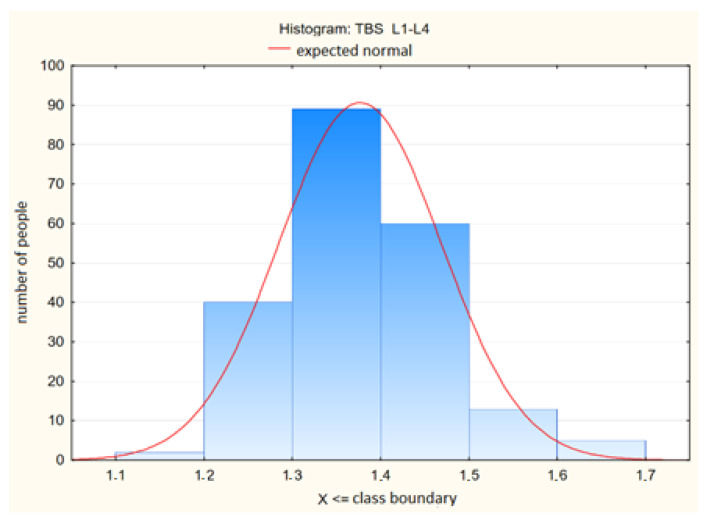
Trabecular Bone Score (TBS L1—L4) Values in the Lumbar Spine Among Study Participants.

The distributions of the bone mineral density of the lumbar spine and the values of the trabecular bone score in this spine are shown in Table 2. and Figure 1 and Figure 2, respectively.

Statistical characteristics of fasting glucose and insulin levels, as well as at the 60th and 120th minute during the 75 g glucose load test, are presented in Table 3.

#### 3.1.1. Factors Affecting Bone Mineral Density and Values of the Trabecular Bone Score in the Lumbar Spine—Results of Unidimensional Analysis

**Figure 3:**
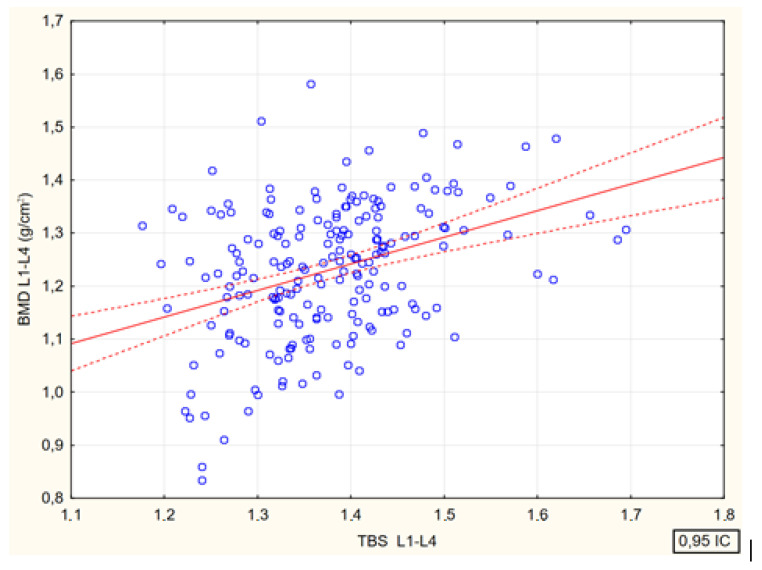
Relationship between lumbar spine trabecular bone score values and bone mineral density in the same segment BMD L1—L4, g/cm^2^ [*p* < 0.001; R = 0.33].

The values of Spearman’s rank correlation coefficients between bone mineral density in the lumbar spine and the values of the trabecular bone strength index in the same were statistically significant (*p* < 0.001). The values of the trabecular bone score showed significantly positive correlations with the values of BMD L1–L4 (g/cm^2^) (R = 0.33); BMD L1–L4 (%) (R = 0.27) and z-score (R = 0.26) (Figure 3).

**Figure 4:**
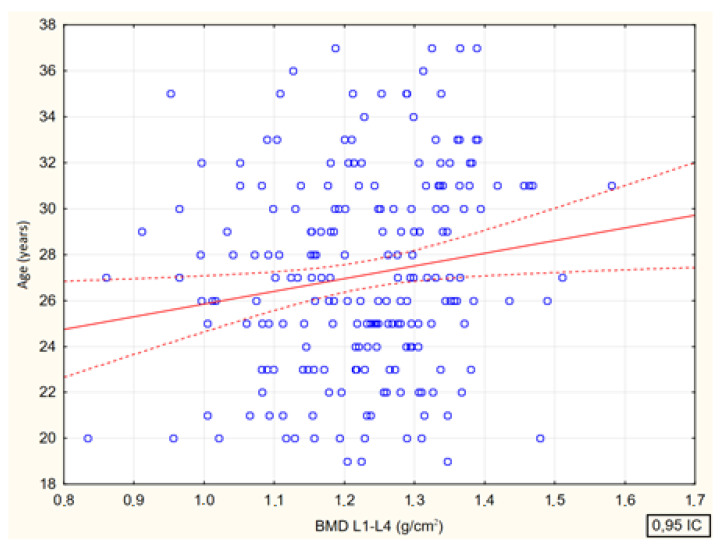
Relationship between lumbar spine bone mineral density (BMD L1—L4, g/cm^2^) and age of study participants [0.026; R = 0.153].

**Figure 5:**
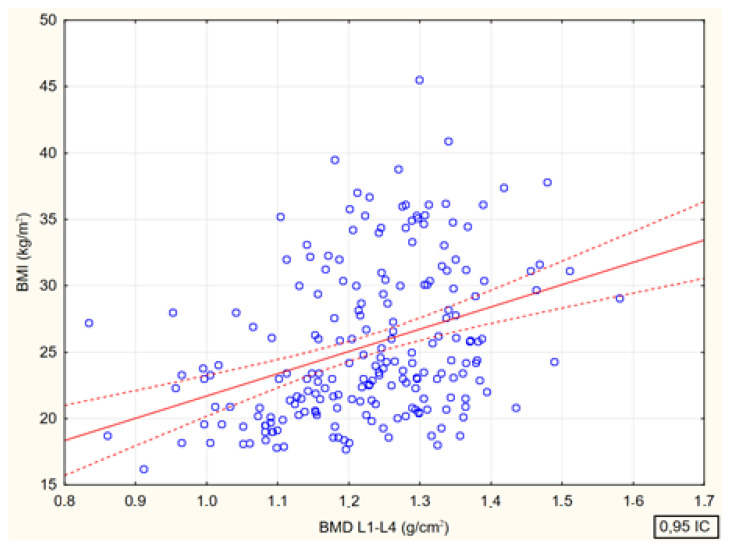
Relationship between lumbar spine bone mineral density (BMD L1–L4, g/cm^2^) and body mass index of study participants [*p* < 0.001; R = 0.39].

**Figure 6:**
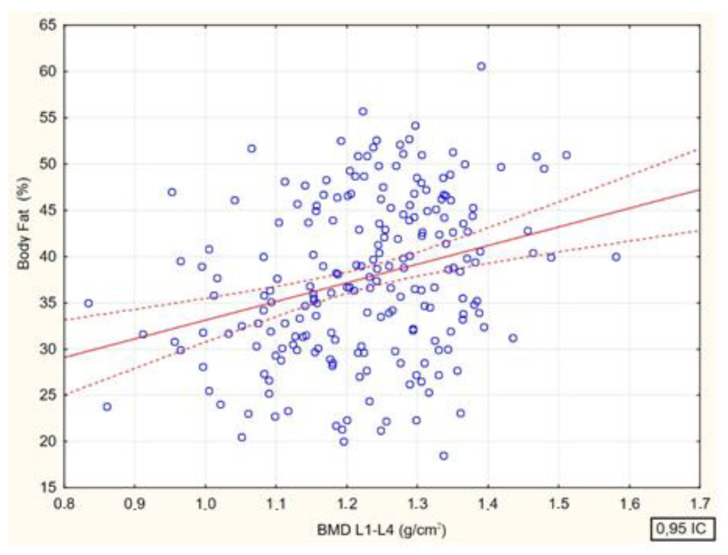
Relationship between lumbar spine bone mineral density (BMD L1—L4, g/cm^2^) and percent body fat of study participants [*p* < 0.000; R = 0.284].

**Figure 7:**
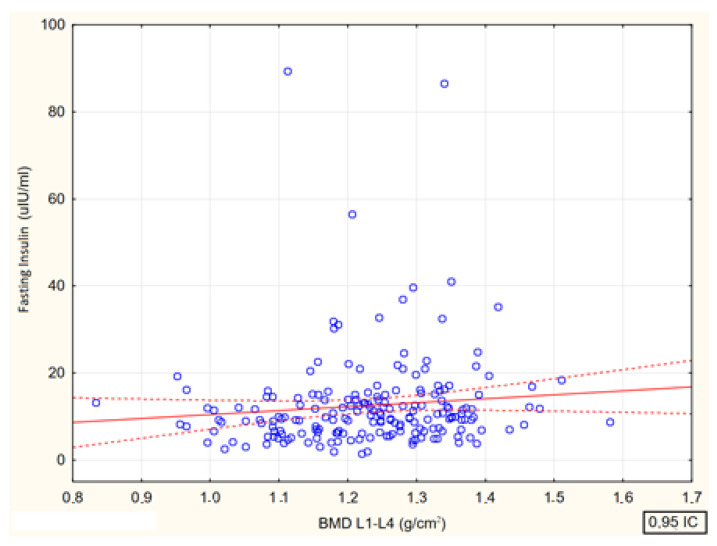
Relationship between lumbar spine bone mineral density (BMD L1—L4, g/cm^2^) and fasting insulin levels in female participants [*p* < 0.016; R = 0.17].

Spearman’s rank correlation coefficients between bone mineral density at the lumbar spine (BMD L1–L4, g/cm^2^) and age (R = 0.15, *p* = 0.026), body mass index (BMI R = 0.39 (*p* < 0.001) and body fat (%) (R = 0.28, (*p* < 0.001) were analyzed. There were significant positive correlations between bone mineral density and all analyzed parameters (Figure 4, Figure 5, Figure 6 and Figure 7).

Spearman’s rank correlation coefficients between lumbar spine bone mineral density (BMD L1–L4, g/cm^2^) and fasting glucose (R = 0.05, *p* = 0.509) and insulin concentrations (R = 0.17, *p* = 0.016), as well as at the 60′ glucose (R = 0.08, *p* = 0.289) and insulin concentrations (R = 0.059, *p* = 0.424) and at the 120′ glucose (R = 0.06, *p* = 0.380) and insulin (R = 0.06, *p* = 0.428) of the 75 g glucose load test showed only a significant positive correlation between bone mineral density and fasting insulin concentrations (Figure 7).

There were significant positive correlations between the values of the TBS and the values of BMI and percent body fat.

**Figure 8:**
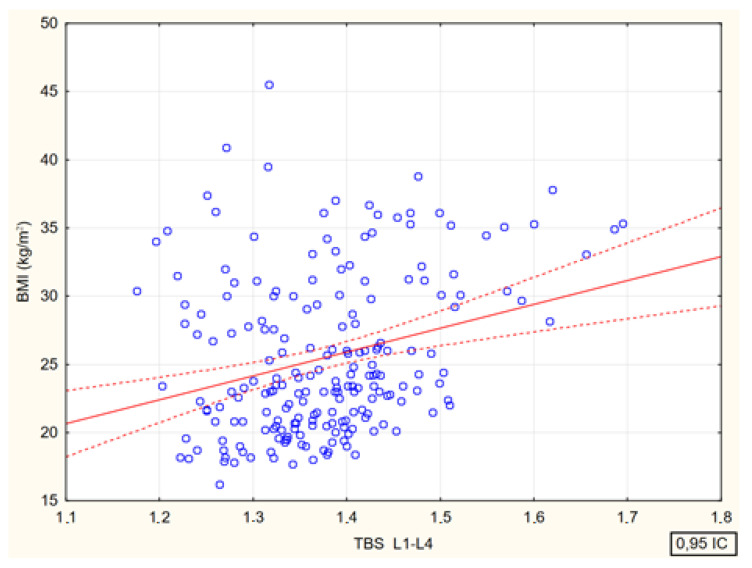
Relationship between values of the trabecular bone score and body mass index values in female study participants [*p* < 0.001; R = 0.28].

**Figure 9:**
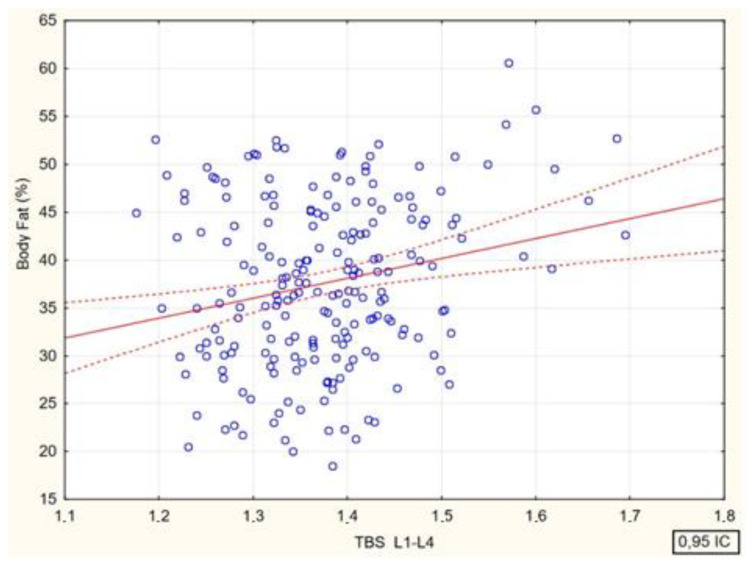
Relationship between the values of the trabecular bone score and percent body fat in female participants [*p* < 0.012; R = 0.18].

Spearman’s rank correlation coefficients between the values of the trabecular bone score at the lumbar spine and age, body mass index, and body fat percentage in the study participants are shown in Figure 8 and Figure 9.

Spearman’s rank correlation coefficients between the values of the lumbar spine’s trabecular bone score and fasting glucose (R = 0.017, *p* = 0.817) and insulin concentrations (R= —0.010, *p* = 0.891), as well as at the glucose 60′ (R= —0.007, *p* = 0.926), insulin 60′ (R= —0.059, *p* = 0.432) and after 120th minutes of the 75 g glucose load test for glucose 120′ (R= —0.039, *p* = 0.595) and insulin (R= −0.058, *p* = 0.427) were not statistically significant. 

#### 3.1.2. Factors Affecting Bone Mineral Density in the Lumbar Spine—Results of Multivariate Analysis

The hypothesis that the trabecular bone score is an independent predictor of bone mineral density in the lumbar spine was therefore verified in the last stage of the study.

In multivariate regression analysis, the following potential predictors of lumbar spine bone mineral density (BMD L1–L4, g/cm^2^) were considered in addition to the girdle bone strength index: patients’ age, percent body fat, body mass index, and fasting insulin levels (Table 4). Of the variables analyzed, the only independent predictor of higher bone mineral density in the lumbar spine appeared to be higher values of the trabecular bone score in the same segment. The proposed model was statistically significant (*p* < 0.001), but explained only about 20% of the variance in the dependent variable (R^2^ = 0.20).

### 3.2. Summary of Results

The results obtained in the univariate analysis were not confirmed in the multivariate analysis, except that the only independent predictor of higher bone mineral density in the lumbar spine was found to be higher values of the trabecular bone index in the same segment. Univariate analysis showed a statistically significant association between BMD and TBS, which was confirmed in multivariate analysis -the only independent predictor of higher bone mineral density in the lumbar spine was found to be higher values of the trabecular bone score in the same segment. In univariate analysis, it was also found that BMD could be influenced by age, body mass index, body fat, and fasting insulin levels. In addition, a relationship between TBS and BMI and body fat percentage is also likely. However, no relationship was observed between TBS score and fasting glucose and insulin in the 75 g glucose load test.

## 4. Discussion

In this study, we checked bone quality based on BMD and TBS in young women diagnosed with functional hypothalamic amenorrhea (FHA). FHA is the term for chronic hypoestrogenism without recognized biological cause. It’s linked to chronic illnesses, overtraining, mental stress, eating disorders, such anorexia nervosa (AN) and undernutrition. Low bone density is a very common finding in these patients. FHA increased the risk of stress or fragility fractures, as well as failure to target height and achieve peak bone mass [37]. A special and most important time of bone quality formation is during puberty and young adulthood. Menstrual disorders can affect these processes, and the longer the absence of menstruation lasts, the lower BMD and bone strength will be [38]. In a study by T. Takeuchi et al. in women with amenorrhea of hypothalamic origin, the effect of glucose loading on serum levels of growth hormone, ovarian and adrenal sex steroid hormones was examined. Gonadotropin levels were unchanged during oral glucose tolerance test (OGTT) in both women with and without FHA. However, positive correlations were found between growth hormone and levels of testosterone, estrogen, dehydroepiandrosterone (DHEAS) during the OGTT test in normal control, stressed and weight loss groups [39]. There are studies indicating a positive relationship between weight gain and bone mineralization due to the stimulating effect of greater mechanical loading on osteogenesis [24]. Mechanical stress stimulates bone formation by decreasing apoptosis and increasing proliferation and differentiation of osteoblasts and osteocytes through the Wnt/β-catenin signaling pathway [4,6].

Because of this, mechanical strain brought on by body weight has given rise to the commonly believed notion that obesity can prevent osteoporosis and bone loss [7,8]. However, further studies have shown that bone mineralization is determined by fat mass, not total body weight or BMI [30,31]. Other studies provided more insight on the subject and showed that adipose tissue is not just a passive reservoir of lipids, but is also a diffuse endocrine gland with region-specific secreted profiles [40]. According to the literature, gynoid fat, or subcutaneous tissue accumulated around the hips, breasts, and thighs, primarily synthesizes pro-osteogenic and anti-osteolytic factors, such as adiponectin, leptin, and aromatase [41,42]. Excessive leptin secretion or reduced adiponectin production by adipocytes in obesity may also directly affect bone formation or indirectly affect bone resorption [28,29,30,43]. Then, pro-inflammatory cytokines (such as TNF-alpha and IL-6) and cell adhesion molecules (like ICAM1 and E-selectin) that promote bone resorption can be found in visceral adipose tissue and most likely in android (abdominal) fat [35,44,45,46,47].

Comparisons of mineral content (BMC) bone density (BMD) and spongy bone (TBS) were studied in adolescents with obesity and extreme obesity. The cross-sectional study included 154 adolescents (12–15 years old, 62% of whom were female) who were classified as having obesity group (OG), (95th–99th percentile) or extreme obesity group (EOG), (>99th percentile). The authors showed that there were no significant gender differences for BMC and BMD measurements, while TBS was lower in EOG compared to OG in both genders in univariate analysis. Thus, extreme obesity affected bone mineralization and was documented by reduced lumbar spine TBS values in adolescents of both sexes [48]. Indirect evidence for this important role of adipose tissue is provided by the results of a spongiosa bone study in adolescent girls with anorexia nervosa (AN). A study by LevyShraga Y et al. retrospectively evaluated 208 adolescent females (mean age 15.6 ± 1.8 years) hospitalized for AN. The mean TBS value was 1.308 ± 0.083, which was lower than values previously described in healthy adolescents, which is ≥1.35 (*p* < 0.001). The TBS value was significantly correlated with age, body weight, BMD measurements of the lumbar spine and whole body, bone mineral apparent density (BMAD), and BMAD Z-score [49]. An investigation by Donaldson AA et al. involved 57 AN women between the ages of 11 and 18. The association between TBS of the spine and DXA-measured body composition, pubertal stage, age, height, weight, BMI, and BMD was demonstrated by these authors (*p* < 0.05). The researchers concluded that the TBS value is evidence of degradation of bone microarchitecture, so it is a novel tool that captures another dimension of bone health in adolescents with AN [50]. Univariate analysis in our study showed that TBS correlates with the aforementioned independent predictors, namely BMI and total body fat. In our study, the number of parameters that influenced BMD in univariate analysis was significantly higher than that of TBS. In addition to BMI and total body fat volume, BMD also correlated positively with fasting insulin. Poyoroznyuk et al. investigated the relationship between lumbar spine, femoral neck BMD, TBS and body mass index in postmenopausal women with osteoarthiritis of the knee. There was a significant negative correlation between TBS and BMI and a positive correlation between lumbar spine BMD and BMI [51]. Visceral adipose tissue shows a high expression of visfatin, which is responsible for stimulating osteoblast proliferation and inhibits osteoclast formation. Retinol-binding protein 4 (RBP-4) is a retinol transporter and is associated with changes in insulin sensitivity. A positive relationship between BMD and RBP-4 is indicated, while an inverse relationship is indicated between markers of insulin resistance, bone turnover, and current BMD. The effect of visfatin on BMD is without significant effect [52]. Type 2 diabetes (T2D) is associated with an increased risk of fractures, especially of the hip, despite preserved bone mineral density (BMD) [33]. Insulin may promote osteoblast differentiation through elevating levels of osteocalcin [53,54,55]. A study by de Araújo et al. examined the relationship between bone assessment by X-ray absorptiometry to assess bone mineral density and trabecular bone score and Homeostatic Model Assessment of Insulin Resistance (HOMA-IR), visceral adipose tissue, and intrahepatic lipids. The trabecular bone score was found to be negatively associated with marrow adipose tissue, insulin resistance, visceral adipose tissue, and intrahepatic lipid measurements. There was also a negative association between saturated lipids in marrow adipose tissue and barrel bone score [53]. The trabecular bone score and factors affecting TBS were evaluated in the paper by Shah et al. The linear relationship between TBS and BMD and hemoglobin A1c, blood pressure, lipids, and insulin resistance was tested. TBS was significantly lower in adults with type 1 diabetes (T1D) compared to controls (1.42 ± 0.12 vs. 1.44 ± 0.08, *p* = 0.02) after adjusting for age, gender, current smoking status, and lumbar spine BMD, despite no differences in lumbar spine BMD between groups. Components of the metabolic syndrome, including diastolic blood pressure, BMI, triglycerides, and insulin resistance, were negatively correlated with TBS in patients with T1D [54]. A meta-analysis involving 7819 women and men conducted in 2020 demonstrated that type 2 diabetes was associated with a reduction in Trabecular Bone Score (TBS) values compared to the control group. Additionally, individuals in a prediabetic state exhibited significantly lower TBS [55]. A distinctive attribute of diabetic osteopathy seems to be an atypical skeletal loading with diminished load efficiency while maintaining BMD, partially because of compromised cortical characteristics [33]. Kim et al. studied 1229 men and 1529 women over the age of 50 to examine TBS of the lumbar spine as an indicator of bone deterioration in people with diabetes. In the women’s results, lower lumbar spine TBS scores were noted in women with diabetes than in women without diabetes. TBS was also negatively correlated with glycated hemoglobin, fasting glucose levels, fasting insulin levels and homeostasis model scores for insulin resistance [56]. In a study Goel et al. examined the risk of fracture in men and women aged 20–39 years who were referred for DXA testing. They compared the role of TBS to BMD in estimating fracture risk. The results presented showed that in young adults, low BMD, but not low TBS, was a predictor of the occurrence of a major osteoporotic fracture, making the case for not routinely measuring TBS in young adults [57]. The study by Jose et al. examined bone microarchitecture and bone mineral density in postmenopausal women. Based on the results, they found that the mean BMD (gm/cm^2^) of the femoral neck in obese women was lower compared to age-matched obese postmenopausal controls. Bone microarchitecture was also found to be significantly lower in obese participants compared to the age-matched obese group and the non-obese group [58].

## 5. Conclusions

Univariate analysis showed a statistically significant association between TBS, BMD and BMI and body fat percentage. For insulin and glucose in the 75 g glucose load test, a correlation was only shown for BMD. The results obtained in the univariate analysis were not confirmed in the multivariate analysis, except that the only independent predictor of higher bone mineral density in the lumbar spine was found to be higher values of the trabecular bone index in the same segment. The best diagnostic effect comes from the simultaneous assessment of BMD and TBS, which together can even more accurately predict fracture risk in young women with FSH, especially in women with metabolic disorders.

## 6. Limitations of the Study

Our study also has its limitations. The first potential limitation is the only study group, which is women with FHA, with no control group. We do not present in the study a comparison to a group of people with a metabolic disorder and no menstrual disorders, or without any chronic diseases and no disorders of metabolic parameters. The study group could also be more diverse in terms of anthropometric and laboratory parameters and bone density assessment. There is also a lack of data for lipid metabolism, i.e., measurements of cholesterol, triglycerides, low-density lipoprotein and high-density lipoprotein. In a future similar study, more factors should also be included in the multivariate analysis and a comparison of TBS with metabolic parameters should be made using this analysis. There are 213 patients in the study group. More participants could enable higher statistical power. Acquiring a more extensive group of patients having such characteristics within one investigation center is a difficult undertaking.

## Figures and Tables

**Table 1:** Statistical characteristics of age and body mass index in female study participants.

Variable	n	Mean	SD	Min.	Max.	Median	Q_1_	Q_3_
BMI (kg/m^2^)	213	25.60	5.82	16.22	45.50	23.80	20.90	30.00
Age (years)	213	27.08	4.33	20.00	33.00	27.00	24.00	30.00

n—number of participants; SD—standard deviation; Q_1_—first quartile; Q_3_—third quartile.

**Table 2:** Statistical characteristics of bone mineral density and values of the trabecular bone score at the lumbar spine in the women studied.

Variable	n	Mean	SD	Min.	Max.	Median	Q_1_	Q_3_
BMD z L1–L4 Z-score	213	0.23	0.98	−2.80	2.50	0.30	−0.40	1.00
BMD L1–L4 (g/cm^2^)	213	1.23	0.13	0.83	1.58	1.24	1.15	1.32
TBS L1–L4	213	1.38	0.09	1.18	1.70	1.38	1.32	1.43

n—number of participants; SD—standard deviation; Q_1_—first quartile; Q_3_—third quartile.

**Table 3:** Statistical Characteristics of Fasting Glucose and Insulin Levels, and at 60th and 120th Minute During the 75 g Glucose Load Test.

Variable *n* = 213	Mean	SD	Min.	Max.	Median	Q_1_	Q_3_
Glucose _0′_	86.65	9.72	18.70	122.71	87.00	81.00	92.00
Glucose _60′_	114.43	36.21	11.00	256.00	112.90	87.20	134.20
Insulin _60′_	88.28	72.79	2.35	494.40	65.78	42.22	106.40
Glucose _120′_	93.69	28.53	10.00	203.70	92.00	73.00	109.00
Insulin _120′_	58.78	60.80	4.10	396.60	37.05	24.22	66.20

n—number of participants; SD—standard deviation; Q_1_—first quartile; Q_3_—third quartile.

**Table 4:** Factors affecting lumbar spine bone mineral density (BMD L_1_–L_4_, g/cm^2^), results of multiple regression analysis.

Variable *n* = 213	Beta	Standard Error of Beta	*p*
TBS L_1_–L_4_	0.290	0.076	<0.001
Age (years)	0.132	0.071	0.066
Fat Tissue (%)	−0.040	0.338	0.905
BMI (kg/m^2^)	0.212	0.140	0.130
Insulin _0′_	−0.057	0.086	0.505

Statistical Significance: *p* ≤ 0.05.

## Data Availability

The data presented in this study are available on request from the corresponding authors, M.K and A.Ć., upon reasonable request.
